# Electroacupuncture Alleviates Neuropathic Pain through Regulating miR-206-3p Targeting BDNF after CCI

**DOI:** 10.1155/2022/1489841

**Published:** 2022-06-09

**Authors:** Wenzhan Tu, Jingjing Yue, Xuqing Li, Qiaoyun Wu, Guanhu Yang, Shengcun Li, Qiangsan Sun, Songhe Jiang

**Affiliations:** ^1^Rehabilitation Medicine Center, The Second Affiliated Hospital of Wenzhou Medical University, Wenzhou, Zhejiang, China 325027; ^2^Shandong University, 27 Shanda Nanlu, Jinan, Shandong, China 250100; ^3^Department of Rehabilitation Medicine, The Second Hospital, Cheeloo College of Medicine, Shandong University, 247 Beiyuanda Street, Jinan, Shandong, China 250033

## Abstract

**Background:**

Electroacupuncture (EA) has benefits for neuropathic pain. However, the underlying mechanisms are still unknown. The current study explores the underlying mechanisms of EA in neuropathic pain of chronic constriction injury (CCI) rats. *Material/Methods*. Overall, 126 Sprague-Dawley (200-250 g) rats were divided into nine groups randomly: the sham-operated, CCI, CCI+EA, CCI+sham EA, CCI+NS, CCI+AAV-NC, CCI+AAV-miR-206-3p, CCI+EA+NS, and CCI+EA+AAV-miR-206-3p groups. The animals were sacrificed 14 days postsurgery. Mechanical withdrawal threshold (MWT) and thermal withdrawal latency (TWL) tests were used to determine differences in neurobehavioral manifestations. qPCR, western blotting, and immunofluorescence (IF) were carried out to detect the expression levels of miR-206-3p, BDNF, BAX/Bcl-2, TNF-*α*, and IL-6. Nissl staining was measured to observe morphological changes in neurons. Transmission electron microscopy (TEM) was employed to evaluate microscopic changes in dorsal horn synapses.

**Results:**

Hyperalgesia was reduced markedly by EA in the CCI model. The expression level of miR-206-3p was elevated, whereas the expression levels of BDNF, BAX/Bcl-2, TNF-*α*, and IL-6 were decreased in EA-treated CCI rats. However, a miR-206-3p inhibitor partially abrogated the analgesic effect of EA and resulted in poor behavioral performance and the BDNF, BAX/Bcl-2, TNF-*α*, and IL-6 expression was elevated as well.

**Conclusions:**

EA can relieve neuropathic pain by regulating the miR-206-3p/BDNF pathway, thus exerting anti-inflammatory and antiapoptotic effect.

## 1. Introduction

It is well recognized that injury or disease of the somatosensory system is responsible for the occurrence of neuropathic pain, presenting with allodynia, hyperalgesia, and abnormal pain [1]. It has become a public health problem worldwide due to the long course of the disease, its high incidence, and its tendency to markedly reduce patients' quality of life [2]. However, there are currently no satisfactory treatments for neuropathic pain [3]. Thus, exploring the mechanism of neuropathic pain to provide novel insight into and a basis for the treatment of pain is of importance. Electroacupuncture (EA), as an acupuncture technique that combines modern electrical stimulation with traditional acupuncture, is popular in clinical practice [4–6]. It is well recognized that EA is effective in ameliorating neuropathic pain, causing few side effects exerting and long-lasting analgesic effects, but the underlying mechanism remains to be further explored [5, 6].

Recently, the function of noncoding RNAs in neuropathic pain has become a hot topic, and noncoding RNAs are speculated to be a new therapeutic target [2, 7]. Research suggests that many microRNAs (miRNAs) participate in the regulatory effect of acupuncture on ischaemic stroke, spinal cord injury, depression, and other conditions [8–11]. However, the effect of EA on miRNA-dependent modulators in neuropathic pain is largely undefined [12, 13].

Brain-derived neurotrophic factor (BDNF), as a member of the neurotrophic factor (NTF) family, is a key neuromodulator of the transmission of pain in the peripheral nervous system and central nervous system (CNS) [14, 15]. Some data suggest that BDNF is connected with the regulatory process by which EA relieves neuropathic pain [16]. Furthermore, as a potential target gene, BDNF is negatively regulated by a variety of miRNAs such as miR-30a, miR-206, and miR-1B [13, 17].

miRNAs are endogenous, noncoding RNA with approximately 22 nucleotides in length that are found widely in eukaryotes and prevent mRNA translation and/or promote protein degradation by complementarily binding to specific sites in the 3′-untranslated regions (3′-UTRs) of target genes [4]. Some studies have demonstrated that miRNAs are related to the process of chronic pain (Qiang [3, 18]) and that EA might enhance the repair of peripheral nerve injury (PNI) through regulating miRNAs [13]. Evidence has suggested that an increase in miR-206 expression aids the recovery from neuropathic pain [15]. miR-206-3p has attracted increasing attention, and BDNF is regulated by miR-206-3p via a conserved binding site in its 3′-UTR [15].

It has been well proved that inflammation, apoptosis, and autophagy are associated with the development of neuropathic pain [19–21]. As a key pathological mechanism, neuroinflammation is connected to the activation of glial cells (such as microglia and astrocytes) accompanied by the secretion of proinflammatory cytokines [22, 23]. Some scholars have suggested that EA alleviates neuropathic pain by exerting antiapoptotic and anti-inflammatory effects [24, 25].

Therefore, the current study will further reveal the potential mechanism related to EA treatment in neuropathic pain from the perspective of a gene regulation network with miRNAs as the core. We hypothesized that EA may improve neuropathic pain through the miR-206-3p/BDNF pathway, which is consistent with the secretion of neuroinflammatory cytokines and neuronal apoptosis in spinal dorsal horn. The rats were used to evoke neuropathic pain by establishing a chronic constriction injury (CCI) model and treated with EA. We used the adeno-associated viral (AAV) vectors to determine the function of miR-206-3p in the EA-mediated alleviation of neuropathic pain and further confirmed the existence of a tight correlation between the analgesia effect of EA and the miR-206-3p/BDNF pathway.

## 2. Material and Methods

### 2.1. Animals and Experimental Design

All animal protocols were approved by the Animal Research Committee of Wenzhou Medical University and performed following the guidelines of the Guide for the Care and Use of Laboratory Animals published by the US National Institutes of Health (NIH Publication No. 85-23, revised 1996). In total, 126 adult male Sprague-Dawley rats (200–250 g) were purchased from the Laboratory Animal Center of Wenzhou Medical University. The rats were housed at a constant temperature (22°C–24°C) under a norma12 h light-dark cycle, with free access to food and water.

The rats were randomly allocated to the following nine groups (*n* = 14 each): S group (sham-operated), M group (CCI modelling), ME group (CCI+EA treatment), MSE group (CCI+sham EA), M+NS group (CCI+normal saline), M+AAV-NC group (CCI+negative control virus), M+AAV-miR-206-3p group (CCI+AAV-miR-206-3p), ME+NS group (CCI+normal saline+EA), and ME+AAV-miR-206-3p group (CCI+AAV-miR-206-3p+EA).

### 2.2. CCI Model

Rats were anaesthetized by 2% sodium pentobarbital (30 *mg/kg, i.p.*) and subjected to CCI based on previous methods with modifications [26]. The left sciatic nerve was carefully exposed and tied with four knots loosely. The distance between ligatures was approximately 1 mm, and all ligatures were of the same tightness. Finally, the nerve was returned to its original location after ligation, and the muscle and skin layers were sutured. The sciatic nerve was exposed but not ligated in the sham-operated group.

### 2.3. Mechanical Withdrawal Threshold (MWT)

Mechanical sensitivity was evaluated with Von Frey's method (IITC Life Sciences, California, USA) between 15:00 and –18:00. Briefly, rats were kept in the testing environment for 15 min to allow them to acclimate and become calm. Von Frey stimuli were given to the plantar surface of the rat paw, and the force that elicited paw withdrawal was recorded. The test was repeated 5 times with 5 min interval, and then, the average value was obtained.

### 2.4. Thermal Withdrawal Latency (TWL)

To evaluate thermal hypersensitivity, an A37370 plantar test apparatus (Ugo-Basile, Milan, Italy) was applied. Briefly, the rats were allowed to adapt to the transparent box for 20 min before the test. The plantar surface of the rat paw was irradiated with infrared light generated by an instrument, and the radiant heat source was automatically stopped when the rat lifted its hindpaws. Each test was conducted 5 times at an interval of 5 min for each paw, and the average value was obtained.

### 2.5. EA Treatment

The acupoints applied in the present study are Zusanli (ST-36) and Yanglingquan (GB-34). Rats were fixed with a rat fixator (similar to a fixed vest, which is self-made and patented by our subject). After training for 2-3 days, the rats can lie down stably and receive electroacupuncture stimulation. Rats in the ME and ME+AAV-miR-206-3 groups were treated with EA intervention every 24 h from the 8th day after CCI, for a total of 7 days. The acupuncture needle stimulated electrically lasting for 30 min using a device at a frequency of 2/100 Hz (1.5 mA) (Hans200e, Jisheng Medical Device) and was inserted 2-3 mm deep. Needles were inserted into the same acupoint at a depth of 0.5 mm, but electrical stimulation was not applied in the MSE group.

### 2.6. Virus Construction and Intrathecal (i.t.) Injection

A recombinant adeno-associated virus (rAAV2/8), pAKD-CMVbGlobin-enhanced green fluorescent protein (eGFP)-H1-rno-miR-206-3p blocking (AAV-miR-206-3p) (1.57*E* + 13 *μ*g/ml), was produced by Obio Biotechnology Co., Ltd. (Shanghai, China). As a negative control, recombinant AAV-pAKD-CMVbGlobin-eGFP-H1-shRNA (AAV-NC) (1.11*E* + 13 *μ*g/ml) was constructed. The AAVs were fluorescence labeled with eGFP. Because adeno-associated virus generally needs more than 2 weeks to replicate and stably express, two weeks before CCI, 8 *μ*l AAV-miR-206-3p or AAV-NC was injected intraspinally into the dorsal L5 spinal cord of the rats using a microinjection syringe (33 G, 10 mm) in situ. The rats in the M+NS group were intrathecally injected with an equal volume of saline as a control.

### 2.7. Quantitative Real-Time PCR

Using TRIzol reagent, total RNA was extracted from tissues. 1000 ng of total RNA from each sample was applied to synthesize cDNA of miRNAs and mRNAs with an RT Reagent Kit (RR037A, TaKaRa). Afterwards, the cDNA was diluted with ddH_2_O and prepared for qPCR using the LightCycler 480 system (Roche, Germany) according to instructions of the TB Green Kit (RR820A, TaKaRa). The 2^-*ΔΔ*CT^ method was performed to calculate the relative expression of miR-206-3p and BDNF. The primer sequences used are shown in Table 1 (5′-3′).

### 2.8. Nissl Staining

Spinal cord tissues were fixed with 4% paraformaldehyde (PA) and embedded with paraffin. Paraffin slices were dewaxed, immersed in Nissl staining solution, and dehydrated with ethanol. The slices were then placed in xylene and sealed with neutral gum. Then, the sections were analyzed under a brightfield microscope (Olympus, Tokyo, Japan).

### 2.9. Immunofluorescence (IF)

After the rats were sacrificed with saline perfusion followed by 4% PA, spinal cord tissues were obtained and postfixed in PA lasting 24 h. Next, the tissues were dehydrated in sucrose solutions, embedded in OCT freezing medium, and cut to 5 *μ*m frozen sections. Afterwards, the sections were rewarmed for 30 min, washed with 0.01 M PBS, and blocked with 0.3% Triton X-100. The incubation of rabbit anti-BDNF (1 : 200, DF6387) was performed at 4°C overnight. Subsequently, the sections were thoroughly rinsing in PBS and incubated with secondary antibodies at room temperature for 1 h. DAPI staining was added for 10 min, and a fluorescence microscope was applied to observe and collect the images (Olympus, Tokyo, Japan).

### 2.10. Western Blot Analysis

On postoperative day 14, the lumbar enlargements of spinal cords were obtained and prepared for total protein extraction. The protein was electrophoretically separated with SDS-polyacrylamide gels and transferred onto PVDF membrane. Following a block with 5% skim milk for 2 h, membrane was incubated with primary antibodies for 16-24 h at 4°C. The membrane was rinsed and then incubated with second antibody for 2 h at room temperature. Subsequently, the membrane was imaged, and ImageJ software was used for quantitative analysis. The primary antibodies were as follows: rabbit anti-BDNF (1 : 1000, ab108319), rabbit anti-Bax (1 : 500, AF0120), rabbit anti-Bcl-2 (1 : 500, AF6139), rabbit anti-TNF-*α* (1 : 500, AF7014), rabbit anti-IL-6 (1 : 500, DF6087), and mouse anti-tubulin (1 : 1000, AF7011).

### 2.11. Transmission Electron Microscopy (TEM)

After the rats were anaesthetized, fresh spinal cord tissues were immediately obtained and fixed overnight with 2.5% glutaraldehyde. After washing, the specimens were placed in 1% osmic acid for 1 h and then stained for 2 h (1% uranium acetate) at room temperature. Dehydration of the samples was performed with gradient acetone followed by embedding. Semithin slices and toluidine blue staining were performed for localization analysis, and ultrathin sections were imaged by TEM (Hitachi, Tokyo, Japan).

### 2.12. Statistical Analysis

All data were shown as the mean ± SD. One-way ANOVA and Dunnett's test were applied for multigroup comparisons. Student's *t*-test was applied for evaluation between two experimental groups. The analysis of TWL and MWT was assessed with two-way ANOVA and Bonferroni's post hoc test. SPSS 25.0 statistical software was employed for analysis, and *p* < 0.05 was considered statistically significant.

## 3. Results

### 3.1. EA Treatment Alleviated Hyperalgesia, Reduced Nerve Damage, and Improved Synaptic Plasticity in CCI Rats

**Figure 1:**
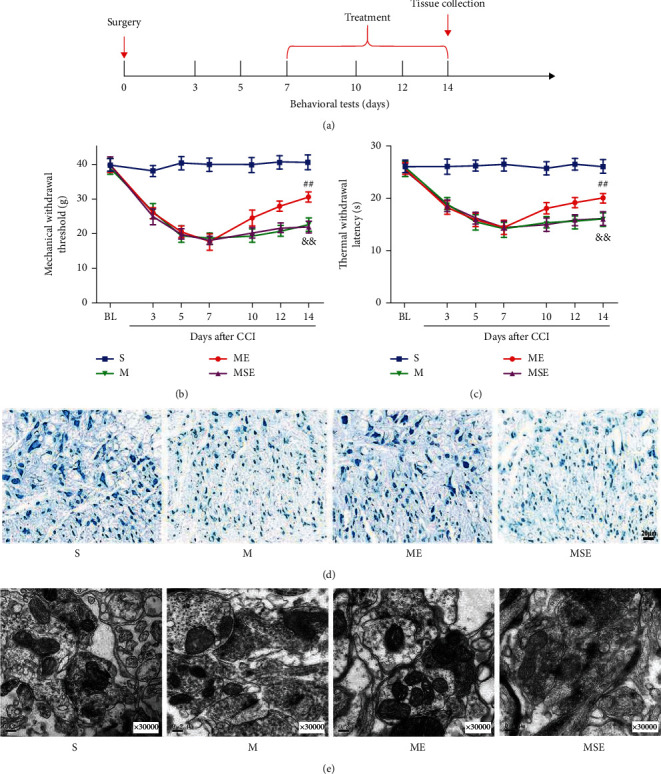
EA treatment alleviated hyperalgesia, reduced nerve damage, and improved synaptic plasticity in CCI rats. (a) Experimental design and timeline. (b, c) Changes in neurobehavioral MWT and TWL values of rats in each group (*n* = 14). (d) Nissl staining to observe the neuron morphology of the spinal dorsal horn (scale bars: 20 *μ*m, 50x). (e) TEM to observe the ultrastructural changes at dorsal horn synapses (0.2 *μ*m, 30000x). ^&&^*p* < 0.01 versus S group; ^##^*p* < 0.01 versus M group.

Both TWL and MWT data (Figures 1(b) and 1(c)) were collected before surgery and 3, 5, 7, 10, 12, and 14 days after surgery. The behavioral value of the rats remarkably decreased after the surgery, except for that in the S group. Both TWL and MWT of the M group were dramatically decreased (*p* < 0.01) as compared with those of the S group at 14 days after CCI. Both TWL and MWT of the ME group were higher than those of the M group (*p* < 0.01). The pain thresholds of the rats in the M and MSE groups were not significantly different (*p* > 0.05).

Nissl staining (Figure 1(d)) indicated that Nissl bodies showed severe injury in the M group, and that this damage was alleviated after EA. TEM (Figure 1(e)) revealed changes in synapses in each group. Notably, the M group revealed abnormal synaptic structures with more synaptic vesicles and narrower synaptic gap than the S group. In addition, the synaptic structure of the ME group was improved, as this group showed the number of synaptic vesicles is fewer and width of synaptic gaps is larger than the M group.

### 3.2. EA Upregulated the Expression of miR-206-3p and Inhibited the Expression of the Target Gene BDNF While Decreasing the Expression of BAX/Bcl-2, IL-6, and TNF-*α* after CCI

**Figure 2:**
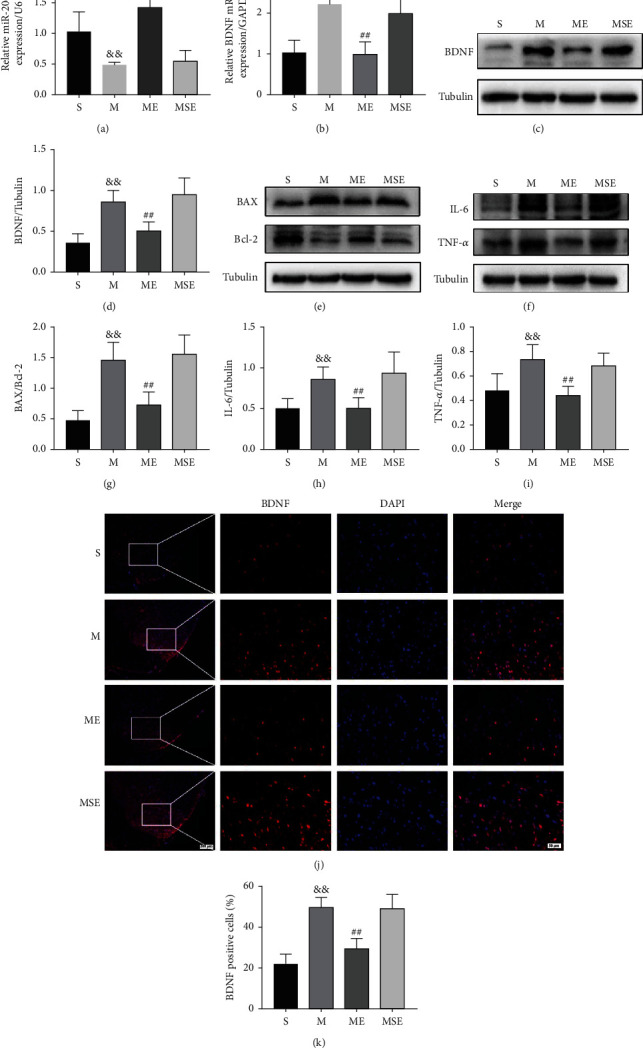
EA upregulated the expression of miR-206-3p and inhibited the expression of the target gene BDNF, while decreasing the expression of BAX/Bcl-2, IL-6, and TNF-*α* after CCI. (a, b) The expression of miR-206-3p and BDNF mRNA (*n* = 5). (c, d) Representative western blots and quantification data of BDNF/GAPDH (*n* = 5). (e–i) Representative western blots and quantification data of BAX/Bcl-2, IL-6/GAPDH, and TNF-*α*/GAPDH (*n* = 5). (j, k) Staining for BDNF-positive (red) cells from the spinal dorsal horn (scale bars: 100 *μ*m 100x; 20 *μ*m, 400x) (*n* = 5). Bars indicate the mean ± SD. ^&&^*p* < 0.01 versus the S group; ^##^*p* < 0.01 versus the M group.

To identify whether miR-206-3p was abnormally expressed in the spinal dorsal horn of CCI rats, the expression levels of miR-206-3p were measured using qPCR. The results of qPCR (Figures 2(a) and 2(b)) showed that the expression level of miR-206-3p in the M group was dramatically reduced (*p* < 0.01), and the expression levels of BDNF mRNA in the M group were dramatically increased (*p* < 0.01), compared with those in the S group. In the ME group, the expression level of miR-206-3p was dramatically higher (*p* < 0.01) and BDNF mRNA was dramatically lower than that in the M group (*p* < 0.01) after 1 week of EA.

The BDNF expression levels were also confirmed by western blotting and IF (Figures 2(c), 2(d), 2(j), and 2(k)), and the results were in line with the mRNA expression levels. Western blot analysis (Figures 2(e)–2(h)) revealed that the expression of BAX/Bcl-2, IL-6, and TNF-*α* in the M group was remarkably increased as compared with that in the S group (BAX/Bcl-2, *p* < 0.01; IL-6, *p* < 0.01; TNF-*α*, *p* < 0.01), whereas the expression of BAX/Bcl-2, IL-6, and TNF-*α* was significantly decreased in the ME group (BAX/Bcl-2, *p* < 0.01; IL-6, *p* < 0.01; TNF-*α*, *p* < 0.01).

### 3.3. A miR-206-3p Inhibitor Aggravated Hyperalgesia in CCI Rats and Damage to Spinal Dorsal Horn Neurons

**Figure 3:**
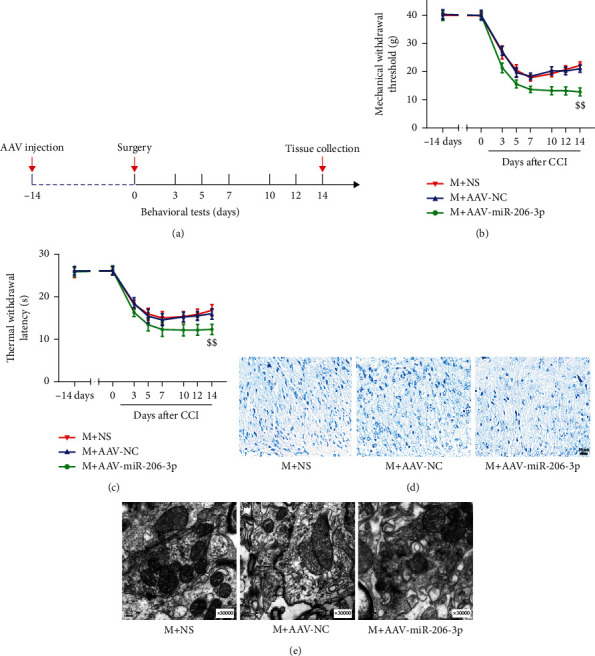
A miR-206-3p inhibitor aggravates hyperalgesia in CCI rats and damage to spinal dorsal horn neurons. (a) Experimental design and timeline. (b, c) MWT and TWL in each group (*n* = 14). (d) Nissl staining images (scale bars: 20 *μ*m, 50x). (e) The ultrastructural changes of synapses in each group (0.2 *μ*m, 30000x). *^$$^p* < 0.01 versus the M+NS group.

To examine the function of miR-206-3p, rats were intrathecally injected with AAV to inhibit miR-206-3p in the lumbar spinal cord 14 days before CCI. The TWL and MWT were measured before surgery (−14 and 0 days) and 3, 5, 7, 10, 12, and 14 days after surgery. Figures 3(b) and 3(c) show that the pain threshold (TWL and MWT) was significantly reduced in all three groups. Both TWL and MWT of the M+AAV-miR-206-3p group were markedly increased on the 14th day after CCI as compared with those of the M+NS group (*p* < 0.01). These data suggested that mechanical and thermal pain hypersensitivity was aggravated by a miR-206-3p inhibitor in CCI rats.

Nissl staining (Figure 3(d)) revealed that neuronal damage was aggravated in the M+AAVmiR-206-3p group than the M+NS group, and vacuole-like changes were observed. TEM (Figure 3(e)) demonstrated that the synapses of neurons in the M+AAV-miR-206-3p group contained more synaptic vesicles and were narrower than those in the M+NS group.

### 3.4. A miR-206-3p Inhibitor Increased the Expression of BDNF, BAX/Bcl-2, IL-6, and TNF-*α* after CCI

**Figure 4:**
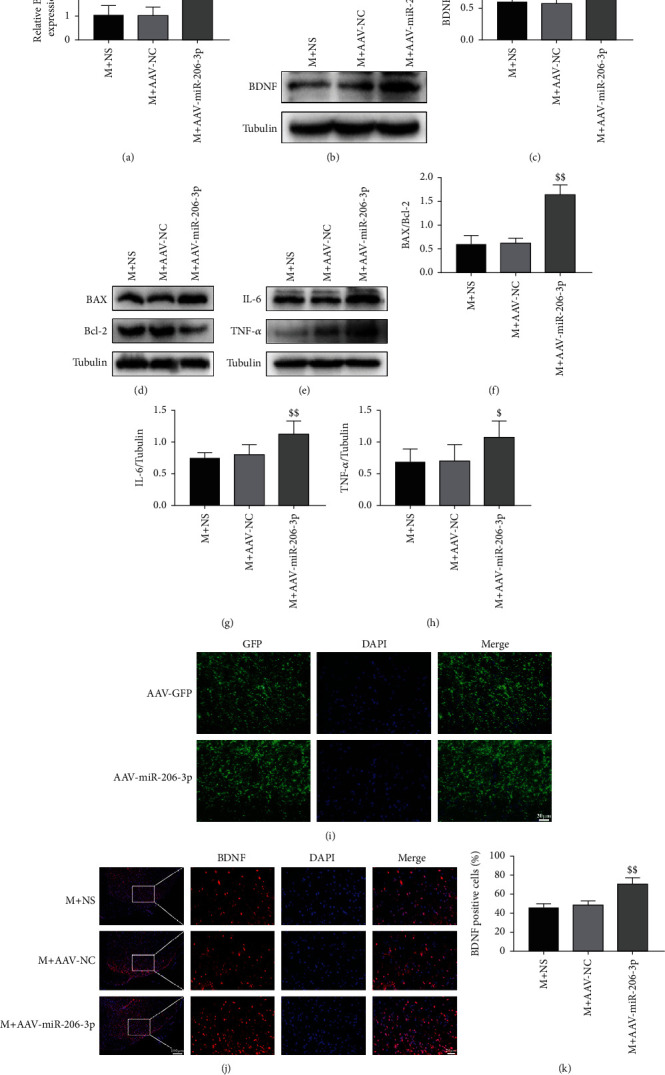
A miR-206-3p inhibitor increased the expression of BDNF, BAX/Bcl-2, IL-6, and TNF-*α* after CCI. (i) Staining for green fluorescent protein- (GFP-) positive cells (scale bars: 20 *μ*m, 400x). (a–c) The mRNA and protein expression of BDNF/GAPDH (*n* = 5). (j, k) Staining for BDNF-positive (red) cells (scale bars: 100 *μ*m, 100x; 20 *μ*m, 400x) (*n* = 5). (d–g) Representative western blots and quantification data of BAX/Bcl-2, IL-6/GAPDH, and TNF-*α*/GAPDH (*n* = 5). Bars indicate the mean ± SD. $*p* < 0.05 and *^$$^p* < 0.01 versus M+NS group.

IF (Figure 4(i)) showed that, 14 days after injection of eGFP-labelled AAV, the rat lumbar spinal cord was transfected with GFP-AAV-miR-206-3p and GFP-AAV-NC. qPCR and western blot analysis (Figures 4(a)–4(c)) exhibited that the mRNA and protein expression levels of BDNF in the M+AAV-miR-206-3p group were dramatically increased as compared with those in the M+NS group (*p* < 0.01).

IF (Figures 4(j) and 4(k)) again verified that the fluorescence intensity of BDNF in the M+AAV-miR-206-3p group was stronger than that in the M+NS group. Western blot analysis (Figures 4(d)–4(g)) showed that the expression of BAX/Bcl-2, IL-6, and TNF-*α* was dramatically increased in the M+AAV-miR-206-3p group than the M+NS group (BAX/Bcl-2, *p* < 0.01; IL-6, *p* < 0.01; TNF-*α*, *p* < 0.05).

### 3.5. A miR-206-3p Inhibitor Blocked the Therapeutic Effect of EA and Did Not Alleviate Hyperalgesia

**Figure 5:**
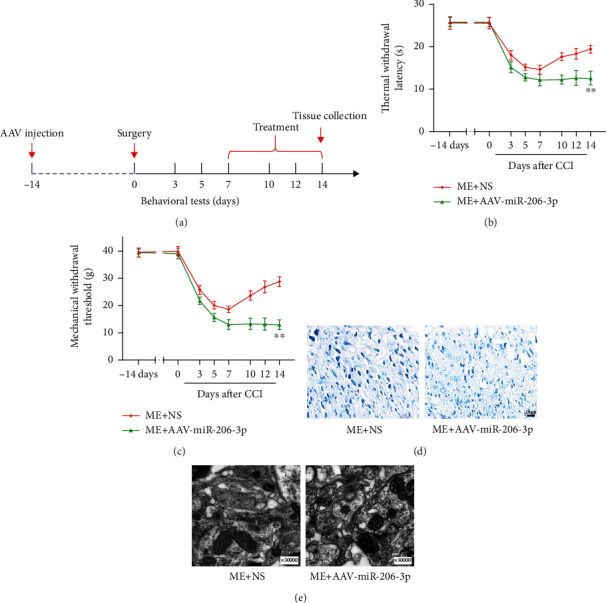
A miR-206-3p inhibitor blocked the therapeutic effect of EA and did not alleviate hyperalgesia. (a) Experimental design and timeline. (b, c) MWT and TWL in each group (*n* = 14). (d) Nissl staining images (scale bars: 20 *μ*m, 50x). (e) The ultrastructural changes of synapses in each group (0.2 *μ*m, 30000x). ^∗∗^*p* < 0.01 versus ME+NS group.

To assess the role miR-206-3p in the effect of EA, we treated CCI rats with EA following AAV injection. The TWL and MWT were both measured before surgery (−14 and 0 days) and 3, 5, 7, 10, 12, and 14 days after surgery. As shown in Figures 5(b) and 5(c), the MWT and TWL of rats in both groups were significantly reduced following surgery. After 1 week of EA, the behavioral scores of the ME+AAV-miR-206-3p group were significantly lower compared with those of the ME+NS group (*p* < 0.01).

Nissl staining (Figure 5(d)) showed that the neurons in the ME+AAV-miR-206-3p group were smaller and shown a more irregular morphology than those in the ME+NS group. TEM (Figure 5(e)) showed that the rats in the ME+AAV-miR-206-3p group had abnormal synapses with narrower synaptic spaces and more synaptic vesicles, while the rats in the ME+NS group had synapses with larger synaptic clefts and fewer synaptic vesicles.

### 3.6. EA Treatment following Injection of a miR-206-3p Inhibitor Did Not Decrease the Expression of BDNF, BAX/Bcl-2, IL-6, and TNF-*α* after CCI

**Figure 6:**
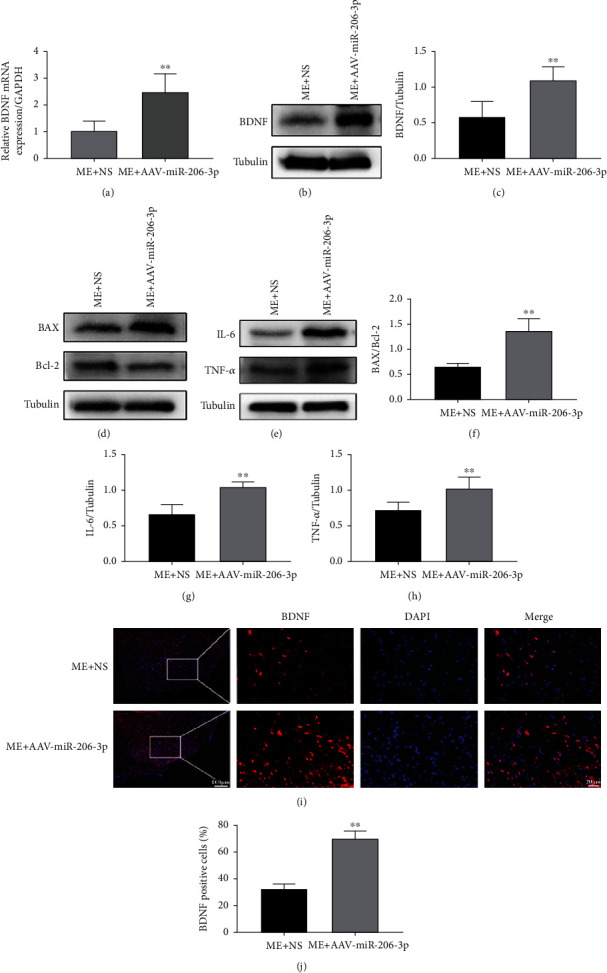
EA treatment following injection of a miR-206-3p inhibitor did not increase the expression of BDNF, BAX/Bcl-2, IL-6, and TNF-*α* after CCI. (a–c) The mRNA and protein expression of BDNF/GAPDH (*n* = 5). (i, j) Staining for BDNF-positive (red) cells (scale bars: 100 *μ*m, 100x; 20 *μ*m, 400x) (*n* = 5). (d–h) Representative western blots and quantification data of BAX/Bcl-2, IL-6/GAPDH, and TNF-*α*/GAPDH (*n* = 5). Bars indicate the mean ± SD. ^∗∗^*p* < 0.01 versus ME+NS group.

The results of qPCR and western blot analysis (Figures 6(a)–6(c)) revealed that the mRNA and protein expression levels of BDNF in the ME+AAV-miR-206-3p group were significantly decreased than those in the ME+NS group (*p* < 0.01). IF (Figures 6(i) and 6(j)) again verified that the fluorescence intensity of BDNF in the ME+AAV-miR-206-3p group was stronger than that in the ME+NS group. Western blot analysis (Figures 6(d)–6(h)) showed that the expression of BAX/Bcl-2, IL-6, and TNF-*α* was dramatically increased in the ME+AAV-miR-206-3p group than the ME+NS group (BAX/Bcl-2, *p* < 0.01; IL-6, *p* < 0.01; TNF-*α*, *p* < 0.01).

## 4. Discussion

**Figure 7:**
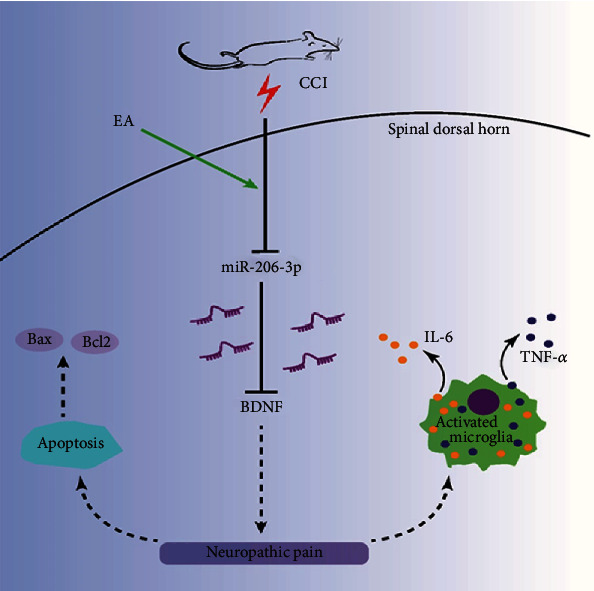
The summary figure for this study. Electroacupuncture exerts analgesic effect by activating miR-206-3P and inhibiting BDNF.

Currently, drugs used to relieve neuropathic pain, including opioids and tricyclic antidepressants, do not meet the clinical needs and commonly induce drug resistance [3, 27]. EA, a simple and efficacious treatment method that is usually used to improve neuropathic pain, has long been the focus of researchers [19, 28]. There are studies indicating that the mechanism underlying the analgesic effect of EA involves a variety of processes, such as peripheral and central nervous-humoural regulation [4]. Many studies have demonstrated that the analgesic effect of EA is associated with the frequency of EA stimulation [29]. In our study, the frequency of EA was 2/100 Hz, which can cause enkephalin, endorphins, and dynorphins to be released simultaneously to achieve better analgesia [4, 29]. Clinically, EA treatment is commonly administered at the Zusanli (ST-36) and Yanglingquan (GB-40) acupoints in patients with lower limb neuropathic pain; and the “Segmental Domination Law” is one of the important clinical acupuncture laws, which is widely used in EA for cervical and lumbar spondylosis [30]. Thus, these two acupoints were selected in this study (Figure 7).

The CCI model used in this study was successfully established by Bennett for the first time, and it was the first model used to evaluate neuropathic pain behavior by the mechanical and thermal pain threshold [26]. This model has been widely used because it is easy to construct and induces long-term and stable pain that is highly similar to neuropathic pain observed in the clinic [3, 31]. Our data indicated that the pain thresholds (TWL and MWT) of rats in each group were significantly decreased after CCI modelling and EA increased these values (Figures 1(b) and 1(c)); this finding is consistent with previous studies.

Neuropathic pain is known to be associated with central sensitization, a process by which the nociceptive signals of neurons at different levels (the spinal dorsal horn, thalamus, and cortex) are gradually enhanced [1, 32]. The spinal dorsal horn plays a central role in pain information transfer and integration, so abnormal excitability and structural remodelling of dorsal horn neurons are crucial for the development of chronic pain [33]. Long-term potentiation (LTP) in the spinal cord is a type of long-term synaptic plasticity and is involved in the central sensitization of pain [23]. Several studies have demonstrated that, after PNI, the release of excitatory neurotransmitters, especially glutamate (Glu), is increased in the spinal dorsal horn, synaptic LTP occurs, and synaptic efficiency is increased [1]. The synapses in the dorsal horn of the spinal cord are key aspects of the connections between neurons, and the increase in their number helps to transmit pain signals between neurons, which may lead to hyperaesthesia during neuropathic pain [23, 34]. The results of TEM indicated that EA alleviated the abnormalities in synapses and that the miR-206-3p inhibitor blocked this effect, by resulting in a smaller synaptic gap and more synaptic vesicles.

During the process of acupuncture-induced analgesia, signals are transferred from acupoints to the CNS, resulting in the spinal cord and brain to release neurotransmitters and neuromodulators (such as opioids, serotonin, and norepinephrine) to suppress pain [4, 35]. Some studies revealed that EA can ameliorate neuropathic pain by inhibiting the activation of microglia and upregulating of BDNF expression in the CCI model [9, 36], which is similar to the results of this study. Moreover, BDNF participates in the formation of spinal cord central sensitization by mediating LTP [33] and is related to synaptic remodelling [13, 15]. miRNAs, as major regulators of gene expression, are known to be associated with the regulation of pain-related networks [2, 37]. Zhao and collaborators confirmed the mechanism by which miRNAs are related to pain in 2010, indicating the importance of miRNAs in inflammatory pain models [28]. Some scholars demonstrated that miRNA-124 and miRNA-146a ameliorate continuous neuropathic pain caused by morphine by targeting Toll-like receptor signalling [38]. As a miRNA with a length of 21 nucleotides, miR-206 has two mature isoforms, namely, miR-206-3p and miR-206-5p [31]. Reports have shown that miR-206 has the capacity to modulate nerve and muscle regeneration [39, 40]. In addition, miR-206 promotes neural remodelling by increasing sympathetic and parasympathetic densities [41]. Moreover, miR-206 regulates the target gene BDNF, which participates in neuropathic pain after PNI [15].

Recently, many findings have linked the effect of acupuncture to miRNA function [4]. Extensive evidence has revealed that acupuncture modulates miRNA-BDNF networks, and notably, the complex features of miRNA-BDNF regulatory networks are consistent with the comprehensive multilevel, multitarget, and multilevel modulatory effects of acupuncture [13]. Studies of depression have shown that EA can target BDNF through miR-206 and miR-155 [13]. In this study, BDNF levels were elevated and miR-206-3p levels were decreased in CCI rats. Moreover, BDNF levels were decreased and miR-206-3p levels were markedly higher after EA treatment. Moreover, the miR-206-3p inhibitor led to the upregulation of BDNF expression in the spinal cord dorsal. Consequently, CCI-induced neuropathic pain was alleviated via EA treatment, which was probably the result of an increase in miR-206-3p expression.

Extensive evidence has indicated that the functions of neurons and surrounding glial cells (including astrocytes and microglia) are often mutually regulated, and the complex communication between them promotes peripheral and central sensitization [22, 42]. The proinflammatory factors (such as IL-6, TNF-*α*, and IL-1*β*) are released by spinal microgliosis and that induces the maintenance of neuropathic pain caused by PNI [43, 44]. The major contribution of IL-6 to nociceptive signalling and central sensitization is well established, and TNF-*α* has been shown to be involved in modulating multiple signalling pathways [22, 45, 46]. Additionally, studies have reported that apoptosis is one programmed form of cell death and is related to the maintenance and occurrence of neuropathic pain [29, 47, 48], and Nissl staining showed that more damaged neurons in the M group were observed than in the S group, which is in accordance with behavioral studies. Furthermore, the BAX gene belongs to the Bcl-2 gene family, which promotes apoptosis, and overexpression of BAX can inhibit the antiapoptotic effect of Bcl-2 and cause cell death [22]. The present study showed that EA treatment led to the low expression of apoptosis-related genes (BAX/Bcl-2) and neuroinflammation markers (IL-6 and TNF-*α*), while a miR-206-3p inhibitor blocked this effect.

Methods such as interference, overexpression, and inhibition have been widely used in the study of miRNA functions [2, 49]. For instance, some scholars have shown that intrathecal injection of a miR-155 inhibitor can decrease the value of mechanical allodynia and thermal hyperalgesia as well as the expression of proinflammatory cytokines (including IL-1*β*, IL-6, and TNF-*α*) in the CCI model remarkably [50]. Viral vectors are commonly applied to control miRNA expression in the nervous system of animal models [2], and AAV vectors are ideal vectors for miRNA research [51]. For instance, Deng and colleagues showed that injecting AAV-SOX10-EGFP into the spinal cord to induce SOX10 overexpression produces mechanical allodynia [52].

In addition, to further clarify whether EA reduces BDNF, BAX/Bcl-2, TNF-*α*, and IL-6 levels in the CCI model by upregulating miR-206-3p expression, we chose to use AAV to inhibit miR-206-3p expression in lumbar enlargement of the spinal cords in CCI rats. We performed studies at day 14 after virus intrathecal injection, and we used IF to demonstrate that the virus was successfully transfected and evenly distributed in cells in the lumbar spinal cord. In contrast to the beneficial effects of EA therapy, the miR-206-3p inhibitor aggravated the neurobehavioral manifestations and changes in neuronal structure and synaptic plasticity in CCI rats, as well as the increased expression levels of BDNF, BAX/Bcl-2, TNF-*α*, and IL-6. It was demonstrated that miR-206-3p expression plays an important role in the analgesic effect of EA in CCI rats and that miR-206-3p may be a potential new target for clinical treatment of neuropathic pain following PNI.

However, in our current study, we were unable to construct a miR-206-3p overexpression AAV vector, which is a limitation of our research. Furthermore, our future work will pay more attention to other miRNAs related to the mechanism underlying the effect of EA as well as other noncoding RNAs. Another limitation of our study is that spinal cord specimens from CCI rats were assessed after only 1 week of EA treatment; thus, we did not assess changes at different time points after EA treatment. More research is needed at different time points to address this limitation in the future. Furthermore, only male rats were used in the current study; thus, the possible impact of sex was ignored.

As shown above, EA therapy at the GB-40 and ST-36 acupoints significantly improved the neurobehavioral manifestations and changes in neuronal structure and synaptic plasticity in CCI rats. Furthermore, EA markedly increased miR-206-3p levels, and this effect was accompanied by decreases in BDNF, BAX/Bcl-2, TNF-*α*, and IL-6 levels in the spinal cord dorsal horn, which were partly blocked by a miR-206-3p inhibitor. Taken together, the current findings suggest that EA has analgesic effects in CCI-induced neuropathic pain, at least partly via miR-206-3p/BDNF regulation.

## 5. Conclusions

In summary, we found that EA alleviates CCI-induced neuropathic pain by promoting miR-206-3p expression and inhibiting BDNF overexpression in the spinal dorsal horn. Besides, we confirmed the role of apoptosis and neuroinflammation in neuropathic pain.

## Figures and Tables

**Table 1:** Primers for rno-miR-206-3p and BDNF.

Gene	Primers
2003rno-miR-206-3p	
Forward	GCGCGTGGAATGTAAGGAAGT
Reverse	AGTGCAGGGTCCGAGGTATT
RT primer	GTCGTATCCAGTGCAGGGTCCGAGGTATTCGCACTGGATACGACCCACAC
U6	
Forward	AGAGAAGATTAGCATGGCCCCTG
Reverse	ATCCAGTGCAGGGTCCGAGG
BDNF	
Forward	GGTTATTTCATACTTCGGTTGC
Reverse	CCCATTCACGCTCTCCAG
GAPDH	
Forward	GACATGCCGCCTGGAAC
Reverse	AGCCCAGGATGCCCTTTAGT

## Data Availability

All datasets generated for this study are available on request to the corresponding author.
